# Next-generation sequencing in the diagnosis of neurobrucellosis: a case series of eight consecutive patients

**DOI:** 10.1186/s12941-023-00596-w

**Published:** 2023-06-02

**Authors:** Lili Yu, Zhenyuan Zhang, Yueli Zou, Xuejiao Qi, Yu Zhang, Kaixuan Bai, Xiaochen Han, Hui Bu

**Affiliations:** Department of Neurology, The Second Hospital of Hebei Medical University, Shijiazhuang, 050000 Hebei People’s Republic of China

**Keywords:** Next-generation sequencing, Neurobrucellosis, Effectiveness, Prompt and specific diagnosis

## Abstract

**Background:**

Neurobrucellosis (NB) presents a challenge for rapid and specific diagnosis. Next-generation sequencing (NGS) of cerebrospinal fluid (CSF) has showed power in detection of causative pathogens, even some infrequent and unexpected pathogens. In this study, we presented 8 cases of NB diagnosed by the NGS of CSF.

**Methods:**

Between August 1, 2018 and September 30, 2020, NGS was used to detect causative pathogens in clinically suspected central nervous system (CNS) infections. Data on demographics, clinical features, and laboratory tests, imaging results and NGS results were collected and reviewed.

**Results:**

Among the presented 8 patients, *Brucella* was rapidly detected using NGS of CSF within 1–4 days, despite those eight patients had variable medical history, disease course, clinical manifestations, laboratory tests and imaging findings. NGS showed the sequence reads corresponded to *Brucella* species were 8 to 448, with genomic coverage of 0.02 to 0.87%. The relative abundance was 0.13% to 82.40% and sequencing depth was 1.06 to 1.24. Consequently, patients were administered with 3 to 6 months of doxycycline, ceftriaxone and rifampicin, double or triple combination, supplemented with symptomatic therapy and were fully recovered except for case 1.

**Conclusion:**

NGS of CSF provides a powerful tool in detection of *Brucella* in a prompt and specific manner, and can be considered for first-line diagnostic use in practice.

## Introduction

Brucellosis is the most prevalent zoonotic infection, with a population-based incidence of about 21.5/100,000 person-years [1] and over 500,000 new cases annually worldwide [2]. Neurobrucellosis (NB) refers to an infection involving the nervous system and accounts for approximately 5% of the Brucellosis cases [3]. Nevertheless, NB can lead to devastating outcomes e.g., sepsis, shock, multiple organ failure and even death, with mortality rate up to 5.5% even the antibiotics are administered.

The main problem with NB is the inadequate- or under-diagnosis, and the primary causes is the non-specific clinical manifestations that NB can presents as encephalitis, meningoencephalitis, polyneuropathy, subarachnoid hemorrhage, radiculitis, myelitis, and psychiatric manifestations et al. [4, 5]. The conventional methods, e.g., microbial culture and serological tests, are limited in wide use for its low sensitivity or specificity, and the need for a long sample-to-answer turn-around time [6, 7]. Polymerase chain reaction (PCR) technique, albeit with a high sensitivity, is mainly dependent on high index of suspicion upon non-specific clinical manifestations; the first-generation sequencing technique aids in diagnosis of NB, but the limited ability to examine nucleic acid sequences at each test makes it impossible widely used in practice [6, 8]. Thus, there is an urgent need for viable new techniques for detecting the causative microorganisms promptly, sensitively and specifically for infections in CNS.

Next-generation sequencing (NGS) is an emerging sequence-based pathogenic microorganism identification technique, and is increasingly used to diagnose the infectious disease of the central nervous system (CNS) [4–6]. Review of the literature showed limited data on NGS for diagnosis of NB, such an acute and serious CNS infectious disease. In 2017, Fan et al. [9] conducted a series of four NB cases, and demonstrated NGS as an effective and quick method for diagnosis of NB, thus allowing prompt treatment with the appropriate antibiotics. From then on, we have not found similar cases or cohort studies, although expanding the use of NGS in practice does warrant more studies with definite conclusions to improve the management of NB.

Given above, in this study, we presented 8 NB cases quickly diagnosed by the NGS of the cerebrospinal fluid (CSF) in the setting of a tertiary referral center in China, reported as follows.

## Material and methods

### Case series

In this study, eight consecutive patients clinically suspected of having NB were admitted to the Department of Neurology of the second Hospital of Hebei Medical University, a tertiary referral center in Hebei province, China, between August 1, 2018 and September 30, 2020. As a part of a research project aimed at using NGS of CSF to detect pathogens in patients with clinical suspected CNS infectious diseases, this study of rarely seen NB cases gained particular attention, and our team prospectively and carefully collected the relevant data during the whole diagnosis and treatment process. The recorded data included demographic characteristics, clinical, radiological and pathogenic findings, and the treatment and outcomes. The protocol of the research project was approved by the Ethics Committee of the Second Hospital of Hebei Medical University (2020-R527), and in accordance with the principles laid down in the Declaration of Helsinki. Before the commencement of this study, each patient involved provided his/her written informed consent to participate.

Patients with following clinical presentations, epidemiology and laboratory test findings were suspected of having NB: 1, history of fever; 2, clinical presentations as altered mental status (defined as decreased or altered level of consciousness, drowsiness or personality changes, coma), epileptic seizure and neck rigidity; 3, CSF findings of leukocytosis, decreased glucose level and rising protein level; 4, epidemiology: recent close contact with livestock, animal products or inhabitants in the affected area before the onset of the disease.

### CSF sample collection

During the lumbar puncture, an additional 2 ml of CSF was taken from each patient and placed into the sterile test tube (SARSTEDT Germany), stored in the refrigerator at − 80 °C within 30 min.

### DNA extraction, DNA libraries construction, and sequencing

The 2 ml CSF samples were then transmitted on dry ice for PACEseq NGS detection (Hugobiotech, Beijing, China). QIAamp DNA Micro kit (Qiagen GmbH, Hilden, Germany) was applied to extract DNA following the manufacturer’s manuals. Then, DNA libraries were constructed using the QIAseq™ Ultralow Input Library Kit for Illumina (Qiagen GmbH, Hilden, Germany). All constructed libraries were assessed for quality using Agilent 2100 Bioanalyzer (Agilent Technologies GmbH, Waldbronn, Germany) and Qubit fluorometer 2.0 (Thermo Fisher Scientific, Waltham, USA). Finally, the qualified DNA libraries were sequenced on an Illumina NextSeq 550 system platform (Illumina, San Diego, USA).

### Bioinformatics analyses

After the sequencing data is offline, adapter, low-complexity, low-quality and duplicated reads and short reads (length < 35 bp) were removed from the raw data of each library to generate high-quality data, using an in-house program. The human host DNA reads were also filtered out by alignment to human reference database (hg38). The clean reads were finally aligned to the Microbial Genome Databases (ftp://ftp.ncbi.nlm.nih.gov/genomes/) using Burrows-Wheeler Aligner software. The reads number and reads per million (RPM) of each detected pathogen was calculated. For detected microbes, including bacteria (*mycobacteria* excluded), fungi (*Cryptococcus* excluded), and parasites, a positive NGS result was given when its coverage ranked top10 of the same kind of microbes and absent in the negative control (“No template” control, NTC) or when its ratio of RPM between sample and NTC (RPMsample/RPMNTC) > 10 if RPMNTC ≠ 0. For viruses, *M. tuberculosis*, and *Cryptococcus*, a positive mNGS result was considered when at least 1 unique read was mapped to species level and absent in NTC or RPMsample/RPMNTC > 5 when RPMNTC ≠ 0.

## Results

### General characteristics and clinical findings

These 8 patients were all male, with a median age of 38 years, ranging 15 to 70 years. Four cases (case 3, 4, 5, 7) were in summer, case 6 and 8 in spring, case 1 in autumn and case 2 in winter. Five cases (case 1, 2, 4, 6, 8) had acute onset, and three (case 3, 5, 7) had chronic course. All had none history of comorbidities except case 1 who had tuberculosis. Four patients (case2, 3, 5, 6) had a history of contact with cattle and sheep (working at raising, contacting or cooking). The general characteristics and clinical manifestations varied greatly between them and were summarized in Table 1**,** and the main complications in Table 2.

**Table 1:** Clinical features of the presented 8 patients with NB

Case	Sex	Age	Disease course (days)	Livestock contact history	Headache	Fever	Hypoesthesia	Back neck pain	Hearing loss	Arthrodynia	Meningeal irritation sign	Unconsci-ousness	Urination disorders	Visual impairment
1	Male	62	30	No	No	Yes	Yes	Yes	No	Yes	No	No	Yes	No
2	Male	32	30	Yes	Yes	Yes	No	No	No	No	No	No	No	No
3	Male	29	180	Yes	No	No	Yes	No	Yes	No	No	No	Yes	Yes
4	Male	15	36	No	Yes	Yes	No	No	No	No	Yes	Yes	No	No
5	Male	28	376	Yes	Yes	Yes	No	No	No	No	No	No	No	No
6	Male	51	15	Yes	Yes	Yes	No	No	No	No	No	No	No	No
7	Male	44	540	No	No	Yes	No	No	No	No	No	No	No	No
8	Male	70	3	No	No	No	Yes	No	No	No	No	No	No	No

**Table 2:** Main complications in patients with neurobrucellosis

Case	Cranial nerves involved	Polyneuropathy	Meningitis	Encephalitis	Myelitis	Cerebrovascular disease
1	No	No	No	No	Yes	No
2	No	No	Yes	No	No	No
3	No	Yes	No	No	No	No
4	No	No	Yes	Yes	No	No
5	No	No	Yes	No	No	No
6	No	No	Yes	No	No	Yes
7	No	Yes	Yes	Yes	No	No
8	No	No	No	No	No	Yes

### Laboratory tests

HIV antibody, syphilis antibody and TORCH tests showed negative, also the cerebrospinal fluid (CSF) assays for acid-fast staining, smear and culture, among these 8 patients. Five patients (case 1, 2, 5, 6, 7) had increased cranial pressure (250 to > 330 mmH_2_O). Routine CSF examination showed elevated white blood cell (WBC) count (68 to 442 × 10^9^/L), with mononuclear cells in absolute predominance (75–90%), elevated protein level (0.8–4.7 g/L) in all except for case 8; reduced glucose in case 1, 2, 4 and 7 (0.91 to 2.40 mmol/L). CSF cytology analysis was performed in six cases, with lymphocytic predominance in case 3, 4, 5 and 7 and no cytological abnormalities in case 8. CSF culture showed negative results for all the 8 cases.

Both Rose Bengal Plate Test (RBPT) and standard agglutination test (SAT) were positive in case 1, 3 and 7, RBPT positive in case 4, and SAT positive in case 6. Only in case 8, the blood culture identified the *Brucella melitensis* at the 7th day after sample was sent for culture (Table 3).

**Table 3:** The CSF routine testing and culture, blood assays and culture

Case	CSF							Blood		
	Pressure (mmH_2_O)	WBC (cells/μL)	MNL (%)	Protein (g/L)	Glucose (mmol/L)	Cytology	Culture	RBPT	SAT	Culture
1	> 330	100	90	3.33	1.63	29%, lymphocytes; scattered erythrocytes and lytic cells	Negative	Positive	Positive	Negative
2	> 330	170	90	0.8	2.4	No	Negative	Negative	Negative	Negative
3	75	68	90	3.2	0.91	Lymphocytes in predominance	Negative	Positive	Positive	Negative
4	50	442	90	4.7	1.52	Lymphocytes in predominance	Negative	Positive	Negative	Negative
5	> 330	300	90	1.22	3.34	Lymphocytes in predominance	Negative	Negative	Negative	Negative
6	250	200	75	2.4	2.73	Not available	Negative	Negative	Positive	Negative
7	280	320	80	3.66	2.4	Lymphocytes in predominance, 10% neutrophilic granulocyte	Negative	Positive	Positive	Negative
8	140	1	0	0.12	3.69	No abnormalities	Negative	Negative	Negative	Positive

*WBC* white blood cell, *MNL* mononuclear leucocyte, *RBPT* Rose Bengal Plate Test, *STA* standard agglutination test

### Imaging findings

**Fig. 1:**
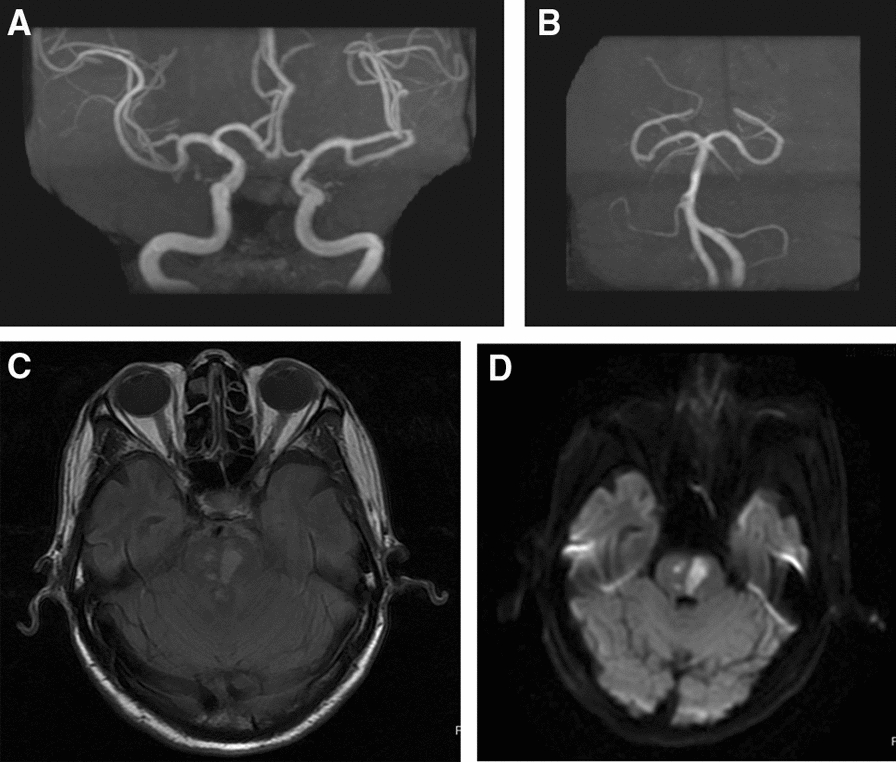
The Brain MR findings showed the mild arteriosclerosis (**A**, **B**), and acute infarction of the brain stem (**C**, **D**) in case 8

**Fig. 2:**
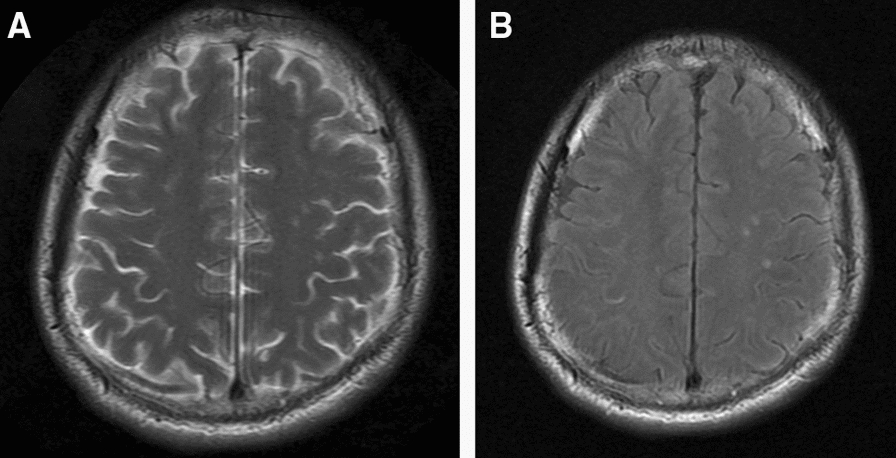
In case 7, the brain MR showed abnormal signal near the right top longitudinal fissure cistern (**A**, **B**)

Brain MR was performed in all 8 cases, revealing the lesion in frontal lobe, lateral ventricle and meninges; cerebral infarction in case 6 and 8. In case 4, abnormal signals was present in the left frontal lobe and around the trigone region of bilateral lateral ventricles, abnormal halo-shaped signals around bilateral lateral ventricles, with slightly widened ventricular system. In case 6, left temporal gaius showed subacute infarction. In case 7, the meninges of bilateral frontoparietal, temporo-occipital lobes appear to be enhanced, with abnormal signals at the right parietal cistern (Fig. 1). Case 8 showed acute cerebral infarction of the brain stem. The remaining 4 patients had not any imaging lesions (focal ischemia, cerebral atrophy, submucosal cyst of maxillary sinus et al.) that were suggestive of *Brucella* infection (Fig. 2).

### NGS results

**Fig. 3:**
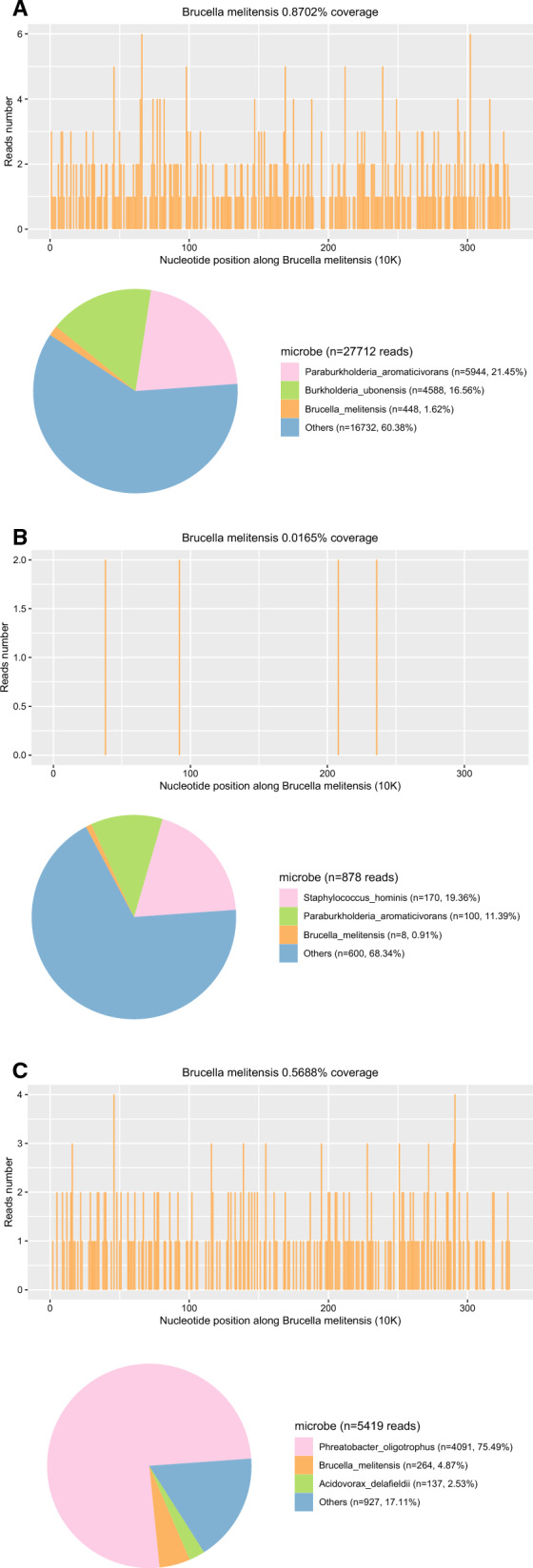
NGS of CSF showing sequences reads of 448, with genome coverage of 0.8702% in case 2 (**A**), 8, and 0.0165% in case 3 (**B**); 137 and 0.5688% in case 7 (**C**), respectively

The time from symptom onset to NGS was 155.3 days on average (17 to 540 days), and after 1 to 4 days the reports returned. A total of 20 species of microorganisms in 13 genera were detected. The proportion of human host nucleic acid sequences was 73.85–99.95% (mean, 95.97%). After excluding background sequences, the sequence reads corresponding to *Brucella* was 8 to 448, with genomic coverage of 0.02 to 0.87%. The relative abundance was 0.13% to 82.40% and sequencing depth was 1.06 to 1.24. These data were presented in Table 4. Figure 3A–C depict sequencing results for case 3, 4 and 5 as examples.

**Table 4:** Next-generation sequencing of CSF for the 8 NB cases

Case	Time from onset to CSF collection (days)	Sample volume (µl)	Specific reads (bp)	Genomic coverage (%)	Relative abundance (%)	Depth (X)	Proportion of human host sequences (%)
1	35	500	266	0.53	3.32	1.15	99.85
2	60	500	448	0.87	1.17	1.2	96.03
3	180	500	8	0.02	0.48	1.1	99.47
4	30	500	86	0.20	82.40	1.08	99.95
5	365	500	116	0.13	3.47	1.24	99.27
6	15	500	126	0.26	7.17	1.11	99.65
7	540	500	264	0.57	3.79	1.06	99.70
8	17	500	72	0.14	0.13	1.19	73.85

*CSF* cerebrospinal fluid, *NB* neurobrucellosis

### Treatment and outcome

After definite diagnosis, all the eight patients were all administered with 3 to 6 months of doxycycline, ceftriaxone and rifampicin, double or triple combination, supplemented with symptomatic therapy. Seven patients had symptoms significantly relieved, and the reexamination of CSF showed substantially decreased WBC and protein level, and were discharged to continue treatment. The condition of the case 1 was not improved, and he gave up continuing treatment and was discharged voluntarily.

## Discussion

In this study, we presented 8 cases of NB in patients who had clinically suspected symptoms or signs, which were definitely and promptly diagnosed with NB using NGS of the CSF. NGS exhibited its superiority over the traditional methods in diagnosis of NB, allowing the early, prompt, high-sensitive and high-specific diagnosis to be realistic. As far as we know, this is the largest case series where NB was diagnosed by NGS of the CSF.

Occupational exposure to animals plays the main factor in the more exposure of males to the incidence of brucellosis [10], which could explain that all 8 patients were male in this study. The clinical manifestations NB were variables, including meningitis, encephalitis, meningoencephalitis, radiculitis, myelitis, peripheral and cranial neuropathies, subarachnoid hemorrhage, and psychiatric manifestations [11, 12], and these were also reflected by our presented cases that meningitis was on case 2, 5 and 6, meningoencephalitis in case 4 and 7, myelitis in case 1, cranial nerve and peripheral neuropathy in case 3, 7 and 8, and cerebral infarction in case 6 and 8. Hence, it seems impossible for use clinical presentations to realize the clear diagnosis.

Currently, diagnosis of NB is primarily dependent on serology, microbial culture, PCR and first-generation sequencing, but various problems exist relating to the lag (generally need for 5–7 days, even up to 1 month for culture of *Brucella*) or inaccuracy [13]. Among them, the positive rate for blood culture was only 28%, and worse still as that for CSF culture, being only 15% [11, 14]. Despite with a higher sensitivity in detection, serological tests lack specificity to distinguish between current and prior infections [15]. The PCR and first-generation sequencing technique appears an ideal technique that the sensitivity and specificity have been substantially improved, but is still not extensively used in practice primarily owing to the low throughput [16]. To summary, the currently used methods or techniques are almost impossible for diagnosis of NB in a simultaneously prompt and precise manner.

Compared with traditional molecular detection methods, meta-genomic NGS has the advantages of high throughput, fast and accurate diagnosis for pathogens, which not only overcomes the limitation of targeted diagnosis by primer design in PCR, but also can obtain drug resistance mutation information and virulence genes to assist diagnosis [17]. All the microorganisms in the samples with DNA as their genetic material can be detected by NGS, and this is the theoretical base for NGS to detect *Brucella*. Due to the fact that the clinical characteristics and CSF findings of NB are generally non- or low-specific, resembling those shown in other CNS infections as tuberculosis, Lyme disease, and Cryptococcus infection [18]. Additionally, even with the same diagnosis of NB, the clinical manifestations and neuroimaging findings are varying dramatically over time [18–20]. In this study, NGS identified *Brucella* within 1 to 4 days, thus allowing prompt and targeted treatment. Furthermore, some of our cases had a long-term course since onset (up to 540 days), but that did not affect the power of NGS in detection of *Brucella*, suggesting its robustness regardless of the course of disease.

There were several case studies that examine the NGS of CSF to diagnose the NB, also showing consistent excellent performance. Fan et al. [18] retrospectively reviewed 4 cases of NB definitely diagnosed by NGS of CSF, where the clinical manifestations were dramatically variable, similar as ours. Jin et al. [21] also reported 3 patients with NB diagnosed by NGS of CSF, and the subsequent appropriate treatments resulted in improved results. In an earlier case report in 2016, Kanokporn [15] reported a case of 11-year old female patient in whom all the various examinations (MRI of the head, blood routine examine, cytologic examination of the CSF, tuberculin skin test, QuantiFERON-TB test) showed negative or undefined findings; only after discharge, the metagenomic sequencing of CSF sample definitely diagnosed the NB and then targeted therapy with doxycycline and rifampin allowed the patient fully recovered. This case deeply impressed the authors by describing the “unexpected answer from meta-genomic NGS”. All of these studies showed the NGS was a pragmatic and reliable technique in diagnosis of *Brucella* infection in central nervous system.

As an emerging technique, NGS faces with a very common problem, contamination, as described in ours and previous studies. This problem is intractable because the sources for contamination are various, such as skin or body flora during lumbar puncture, cross-contamination between samples during preparation of NGS library [18], or the formation of artificial products [15, 22]. In practice, it is necessary to differentiate the actually causative or clinically relevant pathogens from those contaminating or background microorganisms in individual institutions. And thus, researchers or clinicians should be well aware of the common background microorganisms before explaining the results, and during the test with NGS “non-template control” may be a feasible option [18].

There were several limitations to this study. First, the limited number of consecutive patients might affect the results; however, it is unrealistic to conduct a large sample study before NGS technique is extensively used in practice. Even so, the consistent promising findings in this study show the great and particular potential in clinically diagnosis of some infrequent infectious cases of CNS. Second, the single-center design might compromise the generalizability of the results, especially in case of different background microorganisms in individual institutions and contamination. So, the test findings should be comprehensively analyzed and treated, combined with the clinical presentations, laboratory findings and imaging characteristics.

In conclusion, meta-genomic NGS is a promising technique in prompt and precise diagnosis of infectious disease of the CNS, like NB, through our 8 diagnosed consecutive cases using NGS of CSF. This technique aids to guide the targeted therapy and improves the management of NB, leading to an improved prognosis. Despite that, lack of standardized protocol and the contamination from various resources may confound the diagnosis, and the results should be treated comprehensively.

## Data Availability

All the data used during the current study are available from the corresponding author on reasonable request.

## Abbreviations

NB: Neurobrucellosis
NGS: Next-generation sequencing
CSF: Cerebrospinal fluid
CNS: Central nervous system
PCR: Polymerase chain reaction
RPM: Reads per million
